# A concise review of the efficacy of stereotactic radiosurgery in the management of melanoma and renal cell carcinoma brain metastases

**DOI:** 10.1186/1477-7819-10-176

**Published:** 2012-08-29

**Authors:** Peter W Hanson, Ameer L Elaimy, Wayne T Lamoreaux, John J Demakas, Robert K Fairbanks, Alexander R Mackay, Blake Taylor, Barton S Cooke, Sudheer R Thumma, Christopher M Lee

**Affiliations:** 1 Gamma Knife of Spokane, 910 W 5th Ave, Suite 102, Spokane, WA, 99204, USA; 2 Cancer Care Northwest, 910 W 5th Ave, Suite 102, Spokane, WA, 99204, USA; 3 Spokane Brain and Spine, 801 W 5th Ave, Suite 201, Spokane, WA, 99204, USA; 4 MacKay Meyer, MDs, 711 S Cowley St, Suite 201, Spokane, WA, 99024, USA; 5 Gamma Knife of Spokane and Cancer Care Northwest, 601 S. Sherman, Spokane, WA, 99202, USA

## Abstract

Melanoma and renal cell carcinoma have a well-documented tendency to develop metastases to the brain. Treating these lesions has traditionally been problematic, because chemotherapy has difficulty crossing the blood brain barrier and whole brain radiation therapy (WBRT) is a relatively ineffective treatment against these radioresistant tumor histologies. In recent years, stereotactic radiosurgery (SRS) has emerged as an effective and minimally-invasive treatment modality for irradiating either single or multiple intracranial structures in one clinical treatment setting. For this reason, we conducted a review of modern literature analyzing the efficacy of SRS in the management of patients with melanoma and renal cell carcinoma brain metastases. In our analysis we found SRS to be a safe, effective and attractive treatment modality for managing radioresistant brain metastases and highlighted the need for randomized trials comparing WBRT alone vs. SRS alone vs. WBRT plus SRS in treating patients with radioresistant brain metastases.

## Background

The United States faces roughly 170,000 new cases of brain metastases each year, and this number is expected to increase as diagnostic technologies, such as magnetic resonance imaging (MRI), improve and as cancer patients acquire longer survival times [1-3]. The average survival of patients with brain metastases is one to two months with corticosteroid treatment alone, and four to seven months with whole brain radiotherapy (WBRT) alone [4]. The Radiation Therapy Oncology Group (RTOG) recursive partitioning analysis (RPA), which categorizes patients into one of three classes based on the patient’s Karnofsky Performance Score (KPS), age, number of extracranial metastases, and status of primary cancer, with a higher class statistically indicating a worse prognosis, is the most common method for stratifying patients with brain metastases [4]. Out of all new brain metastases cases, approximately 1,200 to 5,100 originate from renal cell carcinoma annually, while roughly 10% originate from melanoma annually [5,6].

Both melanoma and renal cell carcinoma have a well-documented tendency to cause brain metastases. Melanoma represents the third most common primary origin of brain metastases, following non-small-cell lung cancer and breast cancer [7]. The reported clinical occurrence of brain metastases is 8 to 46% in patients diagnosed with melanoma, and autopsy studies found brain metastases in 55 to 75% of these patients [7-10]. In a sizable autopsy series, the occurrence of brain metastases resulting from renal cell carcinoma was reported to be 11% [11,12]. Unfortunately, the expected survival time for these patients is quite low and maximizing these patients’ period of survival and comfort level is of great importance for clinicians. For untreated patients with intracranial melanoma, the median survival time is less than one month without treatment, and for treated patients the median survival time ranges from two to eight months [7,9,13-17]. Renal cell carcinoma patients with brain metastases have a reported mean survival time of three months if left untreated, and with treatment of WBRT the median survival time ranges from two to nine months [12,18-20]. Historically, brain metastases have been treated with WBRT, surgery or both, but increasingly stereotactic radiosurgery (SRS) is emerging as an attractive treatment modality.

SRS is a procedure that employs a high dose of extremely conformal radiation to treat lesions of a small volume in a single treatment session. Prior to treatment, a gadolinium enhanced magnetic resonance imaging is taken of the patient’s head, within a coordinate frame if necessary. A neurosurgeon, radiation oncologist and medical physicist jointly analyze the size, location and shape of the metastases and develop appropriate treatment planning. There are three machine types commonly used for SRS delivery: the linear accelerator (LINAC), the CyberKnife and the Gamma Knife (GK). Studies have shown that the machine type used to apply SRS does not affect treatment outcome [21].

SRS is a desirable and effective treatment option for many patients with newly diagnosed radioresistant brain metastases due to its ability to improve local control, minimally-invasive nature and capability of treating multiple metastases in one setting. SRS has widened the range of treatable patients with radioresistant brain metastases by offering a treatment modality that addresses unresectable brain metastases and results in superior local control when compared to WBRT. We present a concise review of literature analyzing the efficacy of SRS in the management of patients with melanoma and renal cell carcinoma brain metastases.

## Review

Cumulative research shows that SRS is an effective and safe treatment option for patients with radioresistant brain metastases. A recent study by Clarke *et al.*[22] found that GK SRS is a safe and effective treatment for patients with single radioresistant brain metastases, reporting a median survival of 8.1 months. A study by Jensen *et al.*[23] found SRS to be a well-tolerated and well suited treatment modality for patients with radioresistant brain metastases, reporting median survival rates of 7.4 months for patients with intracranial metastatic melanoma and 6.1 months for patients with intracranial metastatic renal cell carcinoma. In a study by Yu *et al.*[14], the overall median survival for patients with intracranial metastatic melanoma was 7 months from treatment of GK SRS, 9.1 months from the occurrence of brain metastasis and 46.7 months from the diagnosis of melanoma. A study by Mori *et al.*[7] found that SRS is an effective and safe treatment modality for patients with intracranial metastatic melanoma, and reported a median survival of seven months following SRS. A study by Noel *et al.*[12] found SRS to be an effective and efficient treatment for brain metastases originating from renal cell carcinoma, reporting an 11-month median survival. A study by Samlowski *et al.*[5] found SRS-based treatment for patients with intracranial metastatic renal cell carcinoma to result in excellent central nervous system (CNS) control, reporting a median survival of 10.1 months after diagnosis of brain metastases. In our experience at Gamma Knife of Spokane, patients with intracranial metastatic melanoma and intracranial metastatic renal cell carcinoma have benefited from stereotactic radiosurgery. We have published that patients with intracranial metastatic melanoma treated with SRS at Gamma Knife of Spokane have a median survival time of 9.7 months [24].

The SRS dose depends on the shape, position and size of the image-guided target. Reported optimal dose ranges are 15 to 22 Gy, with a median of 20 Gy [22]. The RTOG recommends that the maximum tolerated dose is 24 Gy for tumors whose diameters are less than or equal to 20 mm, 18 Gy for tumors 21 to 30 mm in diameter and 15 Gy for tumors 31 to 40 mm in diameter [4]. The surrounding normal brain and outside organs are protected by the conformality of the technique. For example, in GK treatment, only 1/201th of the total radiation dose passes through the body on the way to the target site because there are 201 converging beams. It is only where the 201 beams converge on the metastatic brain tumor that the high dose is deposited. Software programs are also used to help target the radiation at optimal angles and doses to minimize radiation exposure to the rest of the body. Special care is taken when treating metastases around radiosensitive areas, such as the optic nerves or the brainstem.

SRS alone has established itself as an effective treatment modality for the treatment of radioresistant brain metastases, and the addition of WBRT remains controversial. Specifically, WBRT may benefit patients who are younger, have a higher KPS (equal to or greater than 70), or a lower Recursive Partitioning Analysis (RPA) class (1 or 2). A multi-institutional Japanese phase III trial comparing SRS alone vs. SRS plus WBRT in treating patients with intracranial metastases did not find statistically significant differences in both survival and death as a result of neurologic causes between the two treatment arms; however, there was a significantly higher rate of intracranial failures (approximately 50% of patients after six months) in the SRS alone arm [3,25]. One of the arguments against WBRT is the possibility of neurocognitive decline as a side effect, but whether or not WBRT is detrimental to neurocognitive performance is inconclusive and more sensitive tests evaluating neurocognitive performance levels need to be conducted to determine if this is a valid potential side effect [3,26]. However, prospective data from a phase III randomized trial comparing SRS and SRS plus WBRT in patients with one to three brain metastases conducted at M.D. Anderson Cancer Center suggest that post-treatment decline in neurocognitive performance is more likely related to WBRT than to intracranial tumor progression [22]. The addition of WBRT to SRS has shown very limited effectiveness at treating radioresistant histologies, so the inclusion of WBRT should be decided on a case by case basis, based on the patient’s informed decision on whether or not any possible marginal benefits gained from WBRT are worth the downsides of the treatment.

Further randomized trials comparing WBRT alone vs. WBRT plus SRS in treating patients with radioresistant brain metastases are needed because the decision of including WBRT with SRS in treating radioresistant brain metastases is still controversial. These studies are needed to help decisively determine the general standard of treatment for patients with radioresistant brain metastases.

## Conclusions

Melanoma and renal cell carcinoma, considered to be radioresistant histologies, commonly cause brain metastases. SRS has proven to be an effective and safe treatment option for many cases involving these metastases. A prospective randomized trial that assesses the worth of adding WBRT in patients with intracranial metastatic melanoma or intracranial metastatic renal cell carcinoma treated with SRS is necessary. Evidence in the form of data from randomized trials comparing WBRT alone vs. SRS alone vs. WBRT plus SRS in treating patients with radioresistant brain metastases is needed in the future to further define optimal treatment approaches for each patient.

## Abbreviations

CNS: central nervous system; GK: Gamma Knife; KPS: Karnofsky Performance Status; LINAC: linear accelerator; MRI: magnetic resonance imaging; RPA: recursive partitioning analysis; RTOG: radiation therapy oncology group; SRS: stereotactic radiosurgery; WBRT: whole-brain radiation therapy.

## Competing interests

The authors declare no competing financial interests.

## Authors’ contributions

PWH, ALE and CML reviewed relevant literature for this review and drafted the manuscript. WTL, JJD, RFK, ARM, BSC and SRT provided expertise relevant to this review and helped draft the manuscript. All authors read and approved the final manuscript.
